# PReS-FINAL-2333: Long term efficacy of interleukin-1 receptor antagonist (anakinra) in a multicentric cohort of patients affected by idiopathic recurrent pericarditis

**DOI:** 10.1186/1546-0096-11-S2-P323

**Published:** 2013-12-05

**Authors:** M Finetti, A Insalaco, L Cantarini, M D'Alessandro, A Meini, L Breda, M Alessio, P Picco, A Martini, M Gattorno

**Affiliations:** 1 Unit II Pediatrics, G. Gaslini Institute, Genoa, Italy; 2 Ospedale Pediatrico Bambin Gesù, Rome, Italy; 3 Policlinico Le Scotte, Università di Siena, Siena, Italy; 4 Spedali Civili di Brescia, Brescia, Italy; 5 Ospedale Policlinico di Chieti, Chieti, Italy; 6 Università di Napoli Federico II, Naples, Italy

## Introduction

Recurrent pericarditis represents an important complication of acute pericarditis. Therapeutic approach during recurrences consists of NSAID (non-steroidal anti-inflammatory drugs) administration. However steroid is often necessary to control disease flares. IL-1 inhibitors efficacy has been anecdotally described as effective in the control of the disease in steroid-dependent and colchicine-resistant patients.

## Objectives

To evaluate the long term response to treatment with anakinra (IL-1 receptor antagonist) in a multicenter cohort of patients (pts) affected by idiopathic recurrent pericarditis.

## Methods

Fifteen pts (12 pediatrics, 3 adults; M:F = 11:4) followed by 6 national referral centers were enrolled in the study. The mean age was 22 years (range 8-60 yrs); mean age at onset 16 years (5-49 yrs), mean age at the beginning of treatment 19 years (6-56 yrs). All pts received an initial dosage of 1-2 mg/Kg/die. All pts were steroid-dependent and 14 of them had received colchicine. Recurrence was documented in patients who presented typical chest pain and 1 or more of the following signs: fever, rubs, electrocardiographic changes, pericardial effusion, increase of acute phase reactants. Outcomes evaluated in our study were i)response to anakinra, defined as resolution of pericardial symptoms associated to normalization of laboratory-instrumental findings after first administration of the drug; ii)long term remission during IL-1 receptor antagonist regimen defined as absence of relapses during monotherapy; iii)resolution after anakinra discontinuation.

## Results

All pts that received anakinra during active disease (13 pts) presented a dramatic response with a very rapid disappearance of precordial pain, fever, rubs and normalization of acute phase reactants within a few hours from drug administration. Continuous therapy allowed rapid tapering and then discontinuation of steroid, colchicine and NSAID. During continuous daily treatment (mean FU = 12 months, range 5-17 months), no pt presented a relapse of the disease; 14 pts started tapering and 8 of them experienced a relapse (mean time since tapering start to relapse = 9 months, range 2-17 months). In all pts disease flare was successfully and quickly controlled by daily full-dose administration of anakinra, without the requirement of any steroid treatment. A total of 10 flares has been observed in these 8 pts. In 5 pts Anakinra was successfully discontinued after 24 months of treatment (range 17-32 months). The mean time of remission since the withdrawn of the drug is now 12 months (range 2-24 months). At the last follow-up all pts were in remission. Three pts are still receiving daily administration of anakinra as monotherapy. In 7 patients anakinra tapering is ongoing.

## Conclusion

The long term use of anakinra in monotherapy is associated to a persistent control of clinical-laboratoristic-instrumental features of idiopathic recurrent pericarditis. In almost 50% of the patients reactivation of clinical manifestations during anakinra tapering was observed.

## Disclosure of interest

None declared.

